# Anatomy of Cerebro-cerebellar Circuits in Motor Control

**DOI:** 10.1007/s12311-026-02081-4

**Published:** 2026-09-12

**Authors:** Tom J.H. Ruigrok, Zhenyu Gao

**Affiliations:** Department of Neuroscience, Erasmus MC Rotterdam, Rotterdam, The Netherlands

**Keywords:** Cerebellum, Cerebellar nuclei, Cerebral cortex, Basal ganglia, Pontine nuclei, Inferior olive, Tracing, Circuits

## Abstract

Cerebral cortex and cerebellum are intimately connected enabling them to perform their functions in learning, controlling and executing our daily movements. This review aims to provide a summary of both the cortico-cerebellar as well as of the cerebello-cortical routes that are used in their interaction. As such, this review will mostly concentrate on connections that are involved in motor control. Several characteristics of these routes will be accentuated as, for instance, the cortico-cerebellar route involves the cerebellar mossy fiber afferent pathway as well as the climbing fiber pathway, whereas the thalamus seems to be the main output station supplying cerebellar output to the cortex. In addition, the integration of the basal ganglia within the cerebral cortico-cerebellar connections will be highlighted. Modern tracing techniques furthermore supply information on the complex converging and diverging aspects that link the essentially different topical organizations of the cerebral cortex and the cerebellum underscoring the intricate and multifaceted network characteristics of the cerebro-cerebellar system in motor control.

## Introduction

**Fig. 1:**
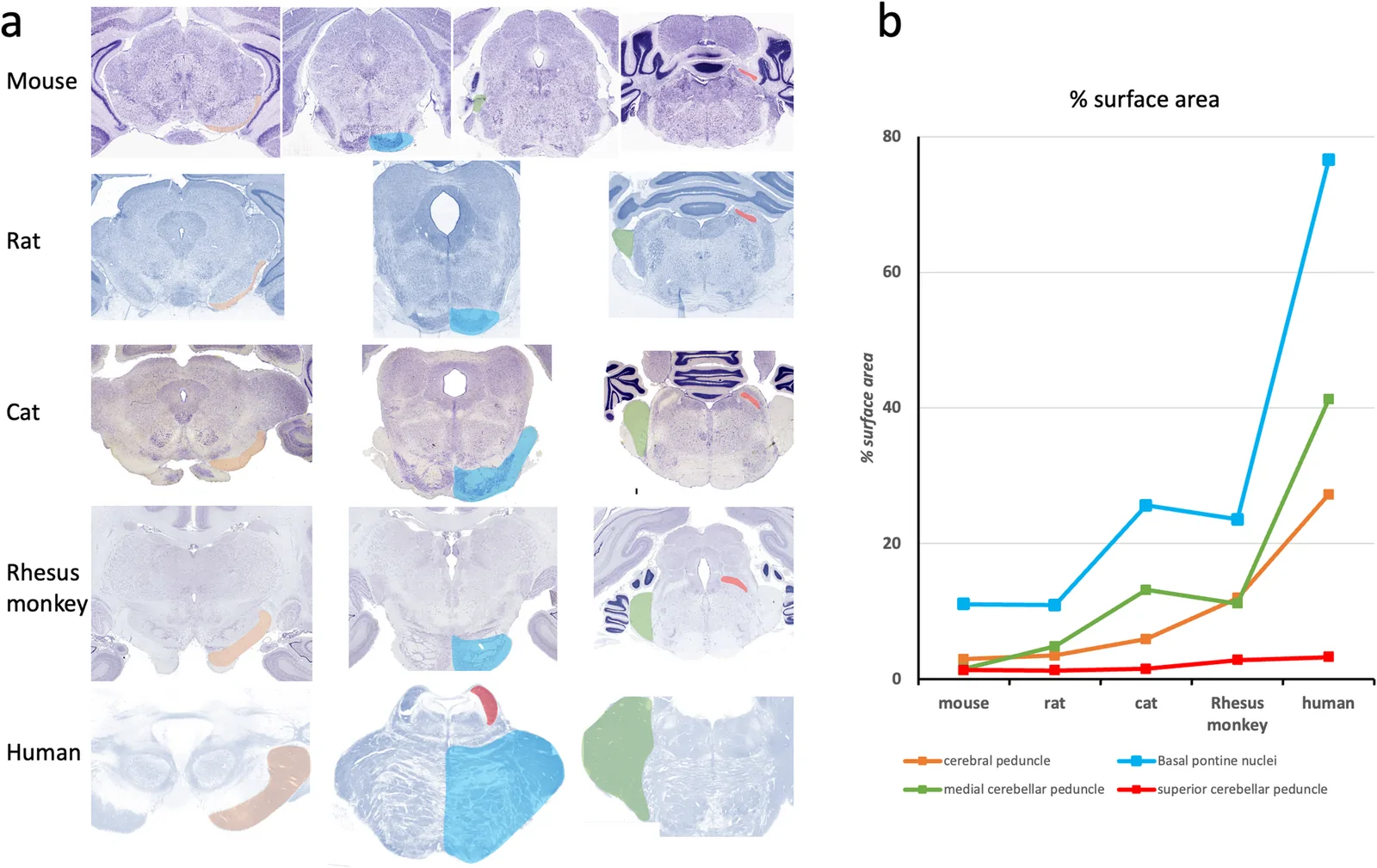
Relative sizes of the cerebro-cerebellar and cerebello-cerebral information routes in different mammals. (**a**) The surface of the cerebral peduncle (orange), basal pontine nuclei (blue), middle cerebellar peduncle (green) and superior cerebellar peduncle (red) are shown in coronal sections of the midbrain and pontine sections of the mouse, rat, cat, rhesus monkey and human brainstem, respectively. (**b**) The percentage of the labeled surface area taken up by these structures is presented relative to the surface area of half of the same section. Note the spectacular increases in size of the cerebral and medial cerebellar peduncles and especially of the basal pontine nuclei in human, whereas the extent of the superior cerebellar peduncle, as a measure of the cerebello-cerebral communication route, remains behind. This figure is only intended to provide an indication of the changes in cerebrocerebellar circuitry. The authors are well-aware that orientation of the brain axis and direction of fibers will be different for the shown species. All sections are Nissl stained, except for the Klüver-Barrera-stained human sections. Mouse sections are taken from Allen Brain Atlas (https://atlas.brain-map.org/), rat and human sections are from the teaching collections of the Erasmus MC, cat and rhesus monkey sections are from the University of Wisconsin and Michigan State Comparative Mammalian Brain Collections (funded by the National Science Foundation and the National Institutes of Health, see https://brainmuseum.org/)

As both cerebrum and cerebellum are absolutely essential for providing timely and adjustable control of learned movements, this review aims to provide an overview of the connections used by the cerebrum and the cerebellum to communicate with each other. Obviously, this connection, to a large extent, is reciprocal as the cerebrum sends information to the cerebellum while, conversely, the cerebellum also instructs the cerebrum. As such the cerebro-cerebello-cerebral (or cerebello-cerebro-cerebellar) circuitry lies at the basis of motor learning, adaptation, execution and control. Yet, already at this stage of describing these routes, two aspects of the circuitry should be highlighted. Firstly, it should be noted that layer V pyramidal cells of large parts of the cerebral cortex contribute to the cerebral peduncle that, although also projecting to a score of other regions, most prominently targets the basal pontine nuclei which forms the origin of the middle cerebellar peduncle that sends information to large parts of the cerebellar cortex. However, the Purkinje cells of the cerebellar cortex first converge on the cerebellar nuclei, the axons of which make up most of the superior cerebellar peduncle that forms the connection with the thalamus. Hence, this reciprocal route usually is less prominent compared to the sheer size of the former two. This quantitative difference in the information stream from cortex to cerebellum versus cerebellum to cortex is especially striking in primates and culminates in human (Fig. 1). Secondly, when describing the connections from the cerebrum to the cerebellum a distinction should be made in routes that supply information by way of the mossy fiber system and information that enters the cerebellum by way of its climbing fibers as both cerebellar afferent systems fulfill functionally quite distinct roles. For the reciprocal connection, the thalamus essentially forms the single most obvious intermediary although other entries carrying cerebellar information into the cerebrum also exist.

Although the authors are well aware that the cerebellum is also involved in non-motor functions such as cognitive, affective and autonomic processes, the related cerebrocerebellar connections, especially in rodents, are not well studied or understood. Therefore, for the sake of clarity and simplicity, we will try to curb our review to connections involved in motor control although we understand that motor and non-motor functions in cerebrocerebellar connections cannot always be clearly separated. Moreover, we will concentrate on data obtained in rodents. Hence, we will first provide a general overview of the cerebro-cerebellar connections detailing both the mossy fiber as well as the climbing fiber routes. Potential other cerebrocerebellar routes that may be important in motor control will be explored. Subsequently, cerebello-cerebral routes will be discussed together with the remarkable collateralization of cerebellar output to brainstem and spinal cord. A vital question in the cerebrocerebellar interaction deals with obvious differences in the cerebral and cerebellar organization. Here, it will be imperative to understand to what extent cerebro-cerebellar and cerebello-cerebral connections are organized along closed or open loops. The intricacies of the cerebro-cerebellar circuit connections should help to evaluate physiological, behavioral and network studies of cerebro-cerebellar interactions.

## Cerebral Connections to the Cerebellum

### Mossy Fibers

As mentioned in the introduction, the communication of the cerebrum to the cerebellum in many animals seems more pronounced than the reverse connection. To a large extent this is brought about by the remarkably robust and extensive fiber pathway linking the cerebral cortex with the basal pontine nuclei, which is a major source of mossy fibers. In rat, it has been long known that layer V pyramidal cells of virtually all cortical areas project to the pontine nuclei, with the highest density of projections coming from the sensorimotor areas [1, 2]. Also, as the largest of the three cerebellar peduncles, the middle cerebellar peduncle carries the axons of the pontine nuclei to the cerebellum where they terminate profusely in the granular layer of large parts of the cerebellar cortex and, to a lesser degree, within the cerebellar nuclei.

**Fig. 2:**
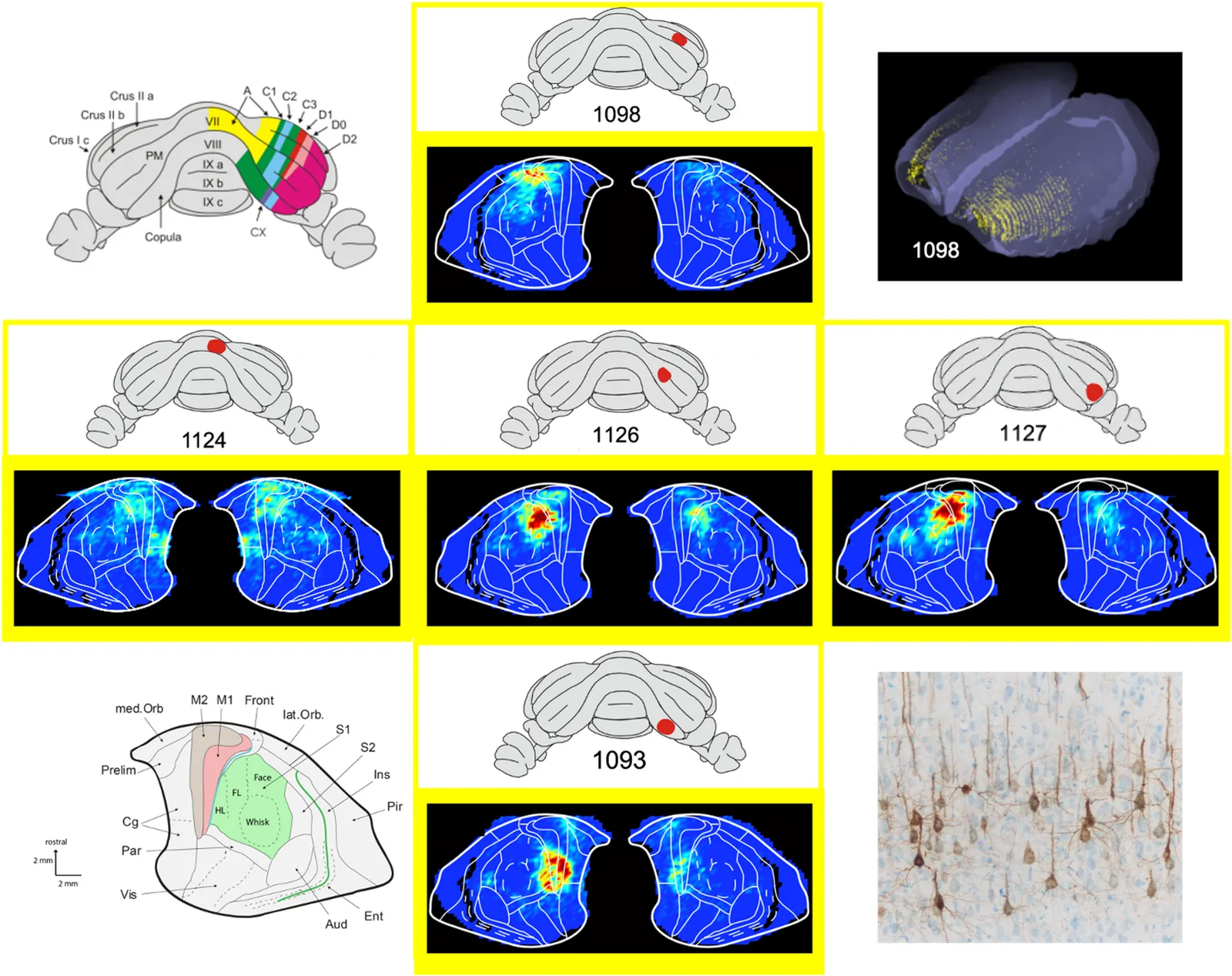
The organization of cerebro-cerebellar connections explored using the retrograde transneuronal infection by rabies virus (RabV) injected at various places of the rat cerebellum. Top left, zonal organization of the posterior cerebellum. These zones are based on the organization of olivo-cortico-nuclear connections. Main figure: five injections are shown, the vertical column shows injections in crus 2B, paramedian lobule and copula pyramidis, respectively, whereas the horizontal row shows three injections in vermal, paravermal and hemispheral parts of lobule VII (paramedian lobule). Below each injection site a diagram showing the distribution of infected layer V pyramidal cells is depicted on a representation of the left and right cerebral cortical surface. Note that all injections results in infection of layer V pyramidal cells in various functional areas that always include patches of M1, M2, S1 and S2 cortical regions. Injections in different lobules result in a shift of the main patch of labeling from a continuous M1, M2, S1 regions from caudal to more rostral caudal regions. Injections ranging from medial to more lateral parts of the same lobule do not change the general pattern significantly (but note the additional patch of labeling in the cingulate cortex after the vermal injection of case 1124). Top right hand inset shows a 3D reconstruction of the cortex showing the location of infected neurons in case 1098. Bottom left hand inset shows the surface representation of the rat cortex indicating the M1, M2 and S1 regions, based on the Paxinos and Watson atlas of the rat brain [147]. Bottom right hand inset shows an example of RabV-infected layer V pyramidal cells in case 1126. Abbreviations bottom left hand inset: Aud, auditory cortex; Cg, cingulate gyrus; Ent, entorhinal cortex; FL, forelimb; Front, frontal area 3; HL, hindlimb; Ins, insula; lat. Orb, lateral orbital cortex; M1, primary motor cortex; M2, secondary motor cortex; med. Orb, medial orbital cortex; Par, parietal cortex; Pir, piriform cortex; Prelim, prelimbic cortex; S1, primary somatosensory cortex; Vis, visual cortex

**Fig. 3:**
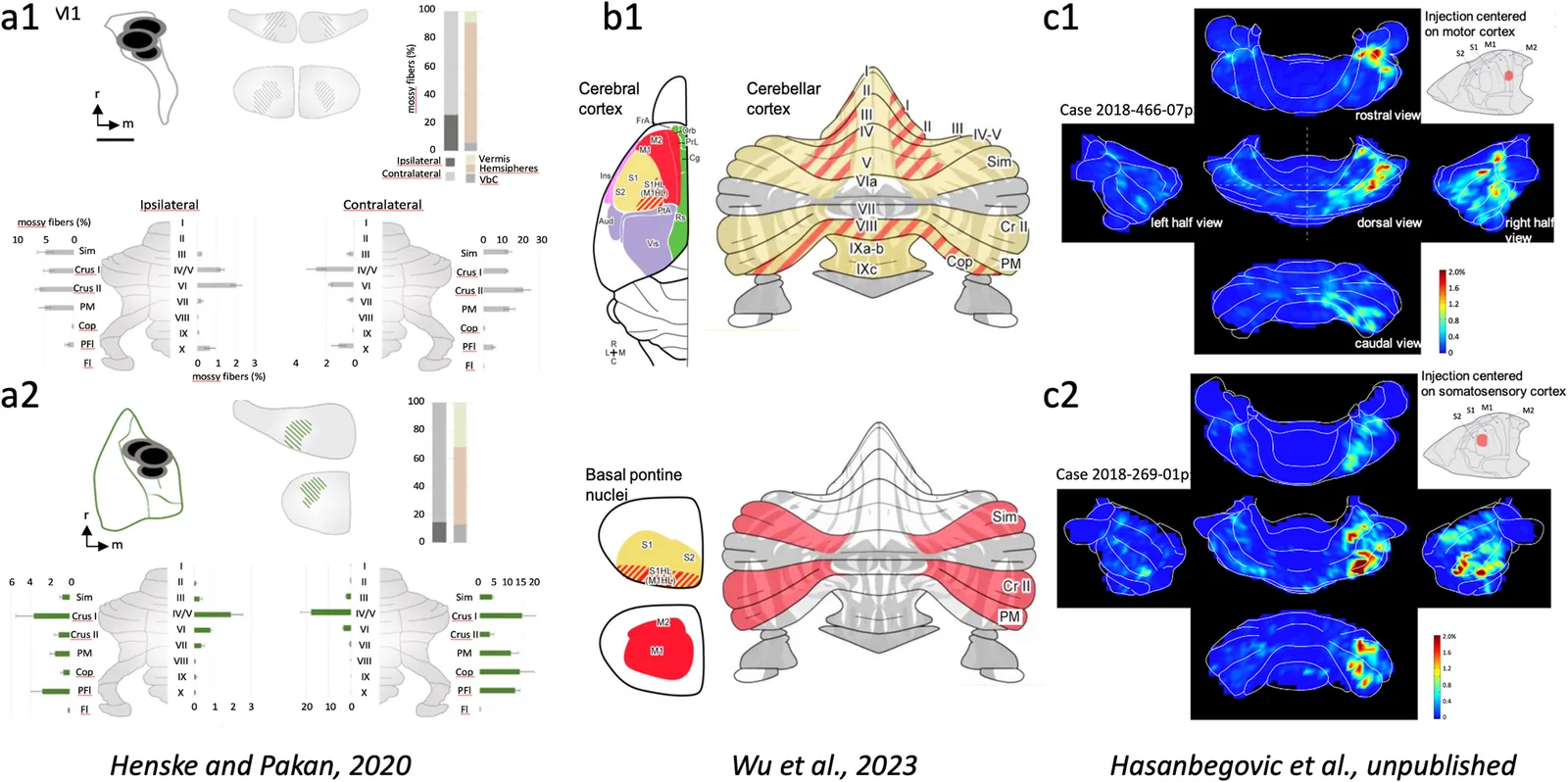
The organization of cerebrocerebellar connections in the mouse explored with antegrade transneuronal infection with AAV1 virus (**a**,** c**) or bidirectional tracing from the basal pontine nuclei (**b**). (**a**) Data from Henschke and Pakan [14] highlights the cortex-wide distribution of the disynaptic cerebro-cerebellar projections originating from a virus injection in either M1 (**a1**) or the limb regions of S1 (**a2**) targeting multiple lobules at their vermal and hemispheral aspects. (**b)** Diagram from Wu et al. [11] showing the projection pattern of the M1/M2 (red), S1/S2 (beige) and S1/M1 hindlimb cortical areas to the basal pontine nuclei and the terminal distribution of mossy fiber rosettes over the cerebellar cortex from these pontine regions. Note the symmetric bilateral aspect of these projections and the large areas of overlap between somatosensory and motor terminal domains. (**c**). Unpublished results from Hasanbegovic, Ruigrok and Gao using a similar technique as used in (**a**) but now showing in color coding the density of plotted mossy fiber rosettes over the cerebellar cortex after an injection in the M1/M2 (top) and S1 (bottom) cortex. Panels show reconstructions of an inverted rostral (top), dorsal (central), caudal (bottom), and left and right lateral views. Injection sites are shown on the top right-hand insets on a diagram of the left cerebral cortex of the mouse [41]

Although the cerebro-ponto-cerebellar connection has long been recognized as both a major and important route for cerebro-cerebellar interaction, its organization has been difficult to describe due to complex diverging and converging connections both in the cerebro-pontine as well as in the ponto-cerebellar projections (for review, see [3]). This made it difficult to describe and understand in what way the functional organization of the cerebral cortex was transposed onto functional aspects of the cerebellum. The functional map of the cerebellar cortex, as based on physiological grounds, has been described as fractionated somatotopy, implying that multiple cerebellar cortical patches, processing mossy fiber information from a particular part of the body are distributed over several cerebellar lobules with varying neighboring patches that process information from surrounding tactile areas [4]. These, thus identified, patches could be matched by stimulating homologous somatosensory cortical areas, implying that spinocerebellar and trigeminocerebellar mossy fiber systems, at least to a degree, overlap with aspects of the cerebro-ponto-cerebellar organization [5]. Indeed, mossy fiber rosettes originating from the external cuneate nucleus may terminate on the same granule cell as mossy fibers from the pontine nuclei [6]. Part of the convergence, however, may also be due to convergence of peripheral and somatosensory cortical inputs within the pontine nuclei [7, 8]. Furthermore, apart from convergence of peripheral and cortical tactile or proprioceptive information, convergence of motor and somatosensory cortices to specified cerebellar cortical areas was demonstrated to specifically depend on converging pontocerebellar mossy fibers [9]. As this notion was corroborated by several studies employing viral and other modern tracing techniques, more light has been shed on the intricacies of the cerebro-cerebellar pathways. Retrograde transneuronal tracing using unmodified rabies virus (RABV) delivered at specified localizations of the posterior cerebellum of the rat showed that information from relatively large areas from the primary and secondary somatosensory cortices converges with information originating from the motor and premotor cortices [10] (Fig. 2). This conclusion was also drawn in a recent study in mice employing bidirectional tracing from injections in the pontine nuclei [11], showing that separated corticopontine projections are directed to highly divergent and consequently overlapping pontocerebellar projections. Indeed, anterograde transneuronal tracing using appropriate adeno-associated viral (AAV) strains [12], also demonstrated the widely distributed nature of cortico-ponto-cerebellar connections [13, 14] (Hasanbegovic et al., unpublished) (Fig. 3). Based on these studies, it can be surmised that for rodents, information from M1/M2 cortical areas reach paravermal and hemispheral parts of the anterior lobe, simple lobule, anterior part of crus 1 and posterior part of crus 2, paramedian lobule and copula pyramidis. Somatosensory cortical information overlaps to a great extent with the motor information, but, in addition, also targets both vermal lobules and hemispheres of these lobules and incorporates the uvula [11].

**Fig. 4:**
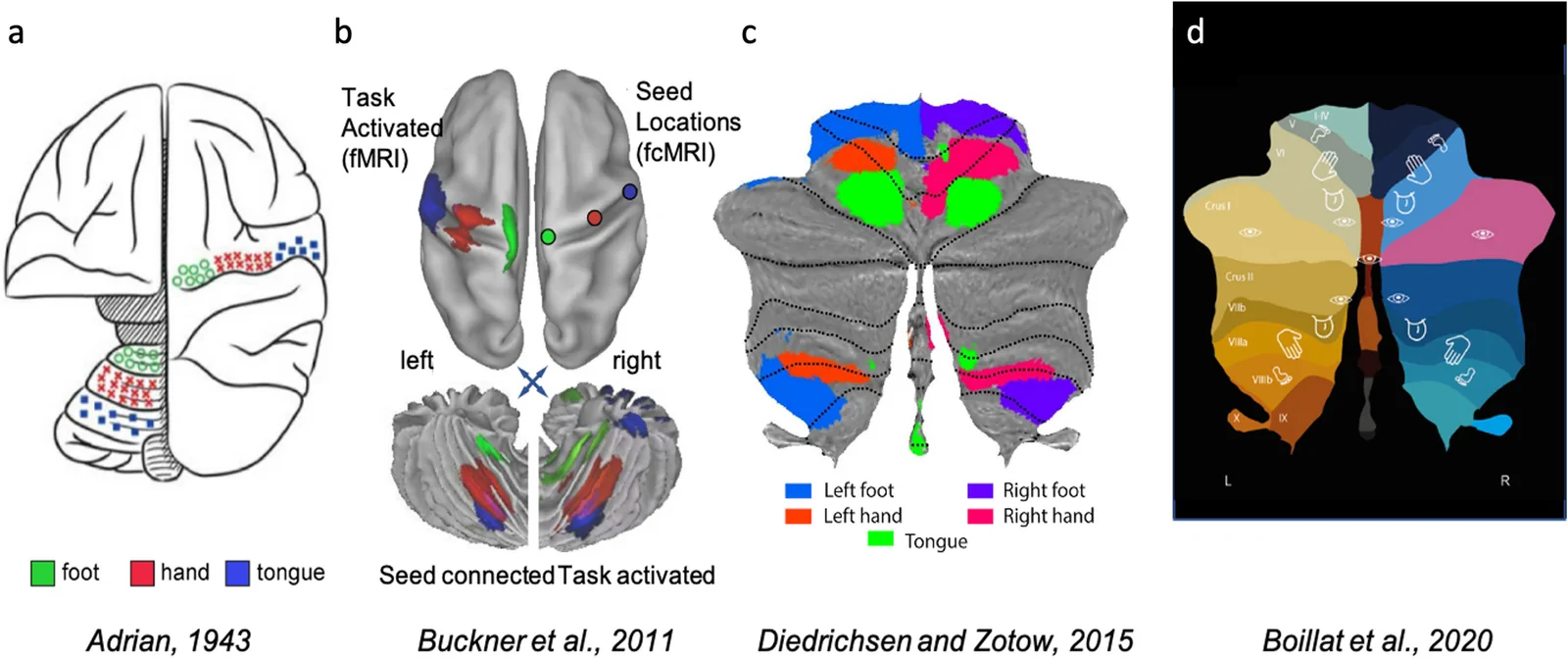
Somatotopic relations between cerebral and cerebellar cortices in monkey (**a**) and human (**b**). (**a**) Somatotopic localization of evoked potentials in the anterior lobe of the monkey cerebellum to electrical stimulation at different positions on the motor cortex. Green, red and blue symbols represent foot, arm and face areas, respectively. Reproduced from Adrian [148]. (**b**) Topography in the human left motor cortex by task-evoked right foot (green), right hand (red) and tongue (blue) movements with functional magnetic resonance imaging (fMRI). The related somatotopic topography can be simultaneously appreciated in an inverted rostral 3D view of the anterior part of the contralateral cerebellar cortex. Seed locations taken at the left primary motor cortex from which the functional connectivity MRI (fcMRI) representations were constructed are shown in the contralateral cerebellar cortex. Note the similarity between the task-based and fcMRI-based estimates of the topographical cortical maps, suggesting that the cerebro-cerebellar connections are active during the execution of the movements. Adapted from Buckner et al. [149]. (**c**) Topography of motor-related areas as depicted on the fMRI-based flatmap of the human cerebellum. Note the inverted representations in the anterior and posterior parts of the ipsilateral cerebellar cortex. After Diedrichsen and Zotow [21]. (**d**) Topography of motor-representations of foot, hand, tongue and eye movements in the same flatmap of the human cerebellar cortex. Note the inverted representations of the body parts in the anterior and posterior cortex. Adapted from Boillat et al. [20]

This concept, to a large extent, is also seen in primates. Cerebellar areas receiving information from cortical sensorimotor areas are found in the anterior lobe/simple lobule and in the paramedian lobule. Within these areas a general and coarse somatotopy can be observed with the leg represented in the anterior-most and posterior-most regions, respectively, with arm and face in respective adjacent regions (for review see: [15]). Note however, that in contrast to rodents, both primate crura are not involved and specifically relate to non-motor functions of the cerebellum [16, 17]. This discrepancy with rodents might relate to a shift in functions but also may reflect distinctions in the relation vermis and hemisphere between rodents and primates [18]. In primates too, he general somatotopy within the cerebellar sensorimotor areas seems to overlap with termination areas of spino- and trigeminocerebellar projections (for review see: [19]). In human, MRI techniques have confirmed this dual representation [20–22] (Fig. 4). In rodents, it has been shown that the anterior and posterior areas, at least partly, receive the same mossy and climbing fiber information [23, 24]. Presently, it is not clear to what extent the anterior and posterior representations are related to developmental constraints or also contribute to different functional aspects of movement control [25]. The anatomy of the mossy fiber organization will be further discussed in relation with the climbing fiber system.

The interaction between cerebral cortico-ponto-cerebellar and spino- and trigeminocerebellar information streams within the granular cell layer and beyond in the molecular and Purkinje cell layers are far from fully understood. This also involves the role of mossy fiber collateral projections to the cerebellar nuclei, which may show a remarkable difference in density between the various sources [26] and which show aspects of learning-induced plasticity [27]. Presently, it is not clear to what extent the cerebellar nuclear terminations of mossy fibers should be regarded as primary or actual collateral branches, i.e., to what extent the nuclear terminations determine the organization and termination of the mossy fiber branches or vice versa [26].

The basal pontine nuclei are not the only intermediary between the cerebrum and the cerebellum. The reticular tegmental nucleus of the pons, lying directly dorsal to the basal pontine nuclei, also supplies most of its efferents to the cerebellum as mossy fibers and receives a prominent input from the cerebral cortex. In rat, most of its afferents arise from layer V neurons of the cingulate, prefrontal, and sensorimotor cortices [28]. The reticular tegmental nucleus, also a target of superior colliculus projections, is usually associated with control of eye movements [29–31]. Alternatively, cerebro-cerebellar information transfer via mossy fibers may occur through the lateral reticular nucleus [32], other reticular nuclei [33], trigeminal complex and dorsal column nuclei [34] or even spinal cord neurons [35].

### Climbing Fibers

A second line of cerebrocerebellar communication makes use of the climbing fibers that emanate from the inferior olive. Each Purkinje cell receives a single branch of a climbing fiber that provides a dense terminal arborization on its dendritic tree [36]. Moreover, collaterals provide fine, sharply bordered, clusters of terminals in the cerebellar nuclei [37]. Relatively sparse direct cortico-olivary connections have been described in rat and cat [38, 39]. In cat, projections mostly emanating from the motor cortex controlling proximal limb and axial musculature are directed to the caudal half of the medial accessory olive. Cortical areas related to the control of proximal forelimb and neck muscles as well as the frontal eye fields direct projections to the transition area between ventral lamella of the principal olive and medial aspect of the dorsal accessory olive [38]. In the rat, a distinction was noted for forelimb and supplementary cortical regions to provide projections to the principal olive, whereas sensory regions direct fibers to the dorsal accessory olive [39].

A more prominent route of cerebrocerebellar communication by way of climbing fibers makes use of an intermediary step at the mesodiencephalic junction. Here, in rodents, the area directly surrounding the retroflex fascicle, approximately midway on its course from the habenula towards the interpeduncular nucleus, forms a well-known source of inferior olivary afferents ánd is known to receive direct projections from layer V pyramidal cells [3, 40, 41]. This area, in rodents sometimes referred to as nucleus, or area, parafascicularis prerubralis (APP) [3, 42], is thought to represent the parvicellular red nucleus of primates and felines [43–45], which also provides a dense innervation of the ipsilateral inferior olive and also receives cortical input, especially from the somatosensory cortex [41, 46, 47]. For the mouse, cortical afferents to the APP are derived in a rough somatotopical fashion with the somatosensory and (pre-)motor cortical regions projecting to the caudal and intermediate APP regions from which efferents reach the rostral part of the medial accessory olive (rMAO) and the ventral leaf of the principal olive (vlPO). Other, more caudally distributed cortical sensory modalities tend to influence the more rostral APP parts, which reach the dorsal leaf of the principal olive (dlPO) [41]. In cat and primates, fibers from the parvicellular red nucleus are bundled in the central tegmental tract, whereas fibers to the MAO follow a more medial course as the medial tegmental tract [19]. The former bundle, together with the red nucleus itself and its target the PO, have expanded enormously in human, highlighting the joint evolutionary growth of cerebrum and cerebellum and underscoring the vital role of cerebro-cerebellar communication by way of the APP/red nucleus and inferior olive [41, 43].

From a functional point of view, an important question is to what extent the cerebro-cerebellar communication line by way of the inferior olive differs from that of the mossy fiber routes. It is well-known that the organization of olivo-cerebellar climbing fibers follows a parasagittal longitudinal pattern, which resembles that of strips of Purkinje cells that project to select divisions of the cerebellar nuclei. This organization is completed by matching projections of olivary climbing fibers to the cerebellar nuclei and projections from the cerebellar nuclei to the inferior olive giving rise to the definition of a number of olivo-cortico-nuclear circuits called modules [26, 48, 49]. These modules adhere to specific chemical boundaries that also indicate differences in physiological processing [50, 51]. On the other hand, as mentioned above, mossy fiber projections are usually described as ‘patchy’ [4]. Yet, pontocerebellar projections have also been described as longitudinal stripes [52]. Indeed, anatomical evidence shows at least a partial adherence of climbing and mossy fiber collateralization patterns [23, 24, 53]. Physiological evidence in rat shows that correspondence of receptive fields of climbing and mossy fiber projections can even be found at the level of single Purkinje cells [54]. Yet, other, rather contrasting, results suggest that Purkinje cells are mostly excited by parallel fibers activated by non-local mossy fibers [55–57]. Activity of local interneurons, responding to similar receptive fields as mossy fibers, also follows sagittal boundaries that adhere to longitudinal patterning (e.g [58]. ; for review see [48]). How the interplay of mossy fiber terminal patterning and activity and climbing fiber patterning and its quite distinct activity, interacts with the cerebellar cortical local circuitry of granule cells, interneurons and Purkinje cells to result in meaningful learning processes is still much debated [48, 59–64].

Apart from the mesodiencephalic junction as an intermediary in cortico-olivary communication a score of other brain areas, such as the zona incerta [65–67], pretectum [68], superior colliculus [69–71], periaqueductal grey [72], dorsal column nuclei [73–75], and olivary projecting neurons in the reticular formation or spinal cord also may provide additional lines for cerebrocerebellar information transfer by way of the inferior olive. In most cases the olivary afferents from these areas, as are the afferents originating from the mesodiencephalic junction are directed to specific parts of the inferior olive implicating them in the control of (parts of) specific modules [for review, see 48].

### Direct Cerebro-cerebellar pathways

Apart from the essentially disynaptic mossy fiber and tri-synaptic climbing fiber cerebro-cerebellar communication routes a direct, monosynaptic, entry of cerebral cortical information has been suggested to exist, either at developmental stages as in cat [76–80] or in the adult as in mice and rat [81, 82] and, very sparsely, in primate [81, 83]. These direct corticocerebellar fibers originated from sensorimotor and prefrontal cortical regions and terminated bilaterally, although mostly ipsilaterally, in the cerebellar nuclei as well as in multiple cortical layers. Presently, it is not known to what extent these direct cerebrocerebellar connections are functionally active [81]. However, in the kitten, at least part of the transient corticocerebellar fibers that collateralize to the spinal cord appear to be mostly silent, which has been suggested to result in their later elimination [77, 78].

### Cerebro-cerebellar Pathways to Cerebellar Nuclei

Most of the precerebellar intermediaries that are targeted by the cerebral cortex not only send projections to the cerebellar cortex but also provide input to the cerebellar nuclei, which serve as the main output station of the cerebellum. Usually, the mossy fibers and nuclear afferents are collaterals as they originate from the same neurons. Although the interplay between these nuclear afferents and the superposed cerebellar corticonuclear loop is far from clear, it should be recognized that the distribution of the nuclear directed afferents and the cortical termination of the related mossy fibers may subserve developmental as well as functional aspects [for review, see 26]. Furthermore, it is clear that the density of the nuclear afferents differs substantially. Nuclear afferents from the basal pontine nuclei are described as rather small and simple, although they may show a tendency to proliferate related to learning [27], whereas afferents from the reticular nucleus of the pons are more robust [84, 85].

In this respect it is interesting to note that the magnocellular part of the red nucleus, origin of the rubrospinal pathway, and receiving input from motor and premotor cortex [47, 86], provides a projection to the cerebellar nuclei without reaching the cerebellar cortex [87, 88]. As this rubrocerebellar projection specifically terminates within the anterior interposed nucleus, which on its turn provides a major excitatory input to the magnocellular red nucleus, this rubro-interposito-rubral loop may act as a prime example of cerebello-precerebellar self-activating, or reverberating, circuits [89].

Cerebellar nuclear afferents from the inferior olive, terminating as a fine varicose network, indefinitely can be seen as branches from the climbing fibers (van der Want et al., 1987). As mentioned earlier, the pattern of termination matches that of the corticonuclear organization of Purkinje cell efferents targeted by the related climbing fibers [49, 90, 91]. As with the collaterals from mossy fiber systems, the density of olivary efferents to the cerebellar nuclear complex is not uniform [92], making it difficult to assess their role in cerebellar functioning [26].

### Modulatory Systems

Apart from mossy and climbing fiber systems it is well known that various monoaminergic and cholinergic systems provide modulatory input to the cerebellum. These systems, at least to some extent, may also be controlled by the cerebral cortex. E.g. cerebellar directed serotonergic fibers, mostly arising from the ventrolateral pontomedullary reticular formation [93], may be targeted by medial prefrontal cortical regions [94]. The same cortical area has also been suggested to influence the locus coeruleus [95], which also sends noradrenergic efferents to the cerebellar cortex [96]. Likewise, dopaminergic input to the cerebellum originating from the ventral tegmental area [97] may be influenced by excitatory fibers from medial prefrontal cortical regions [98, 99]. Cholinergic projections to the cerebellum arise from the pedunculopontine nucleus, which, among other sources of input, is innervated by the (pre-)motor cortex and frontal eye fields [100–102]. Finally, several peptides, such as cholecystokinin, enkephalin and corticotropin releasing factor may colocalize in subpopulations of mossy and climbing fibers [103, 104].

### Other Cerebrocerebellar Pathways

Although the cerebral cortex is the most obvious source for cerebellum-directed information, the basal ganglia, largely part of the cerebrum, also can be seen as involved in communication with the cerebellum [105]. However, we will discuss the connections of the basal ganglia with cerebellum together with the connections of the cerebellum with the basal ganglia in the section on ‘*Cerebellar connections with the basal ganglia’*.

## Cerebellar Connections to the Cerebrum

### Modular Organization of the Cerebellum

**Fig. 5:**
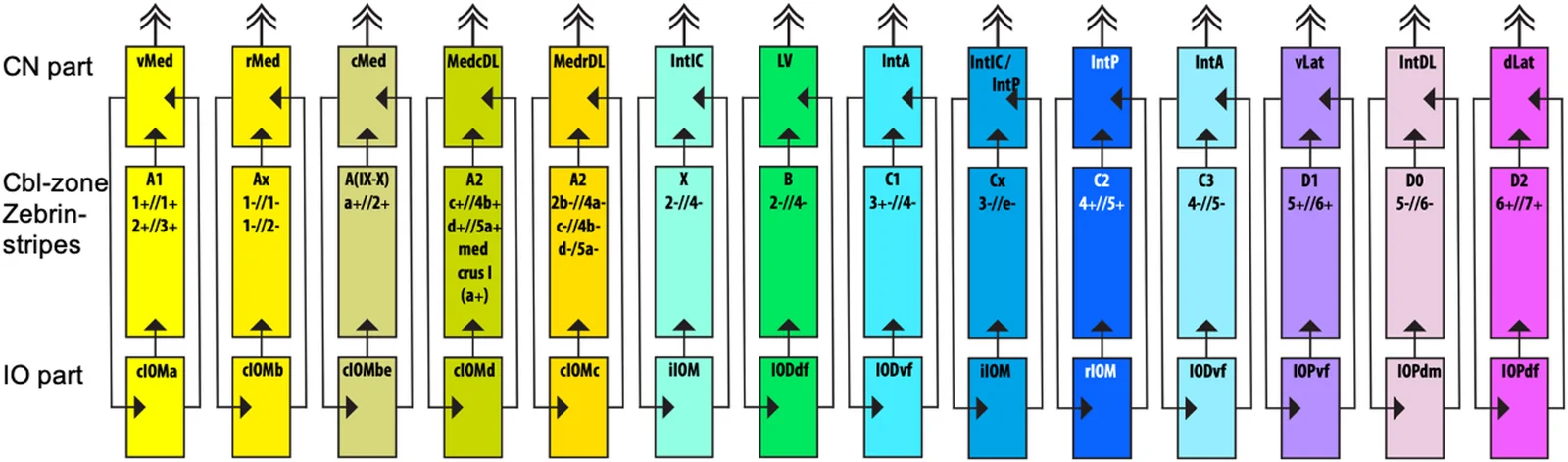
Schematic representation of the olivo-cortico-nuclear interconnectivity. Note that each module, apart from their interconnected parts, also connects to the rest of the brain by their output (double arrowheads). Also note that the cortical part of each module is formed by one or several stripes of either zebrin II + or zebrin II Purkinje cells. The mediolateral order of the represented modules, indicated from left to right in the diagram, is based on the mediolateral position of the CN and is not related to the mediolateral position of the cortical components. Different shades of yellow, blue, and purple refer to Med, Int, and Lat modules, respectively. The green module is related to the lateral vestibular nucleus (LV). Note that modules of the vestibulocerebellum are not indicated in this scheme. Abbreviations. Inferior olive (IO) per module from left to right: cIOMa, group a of caudal medial accessory olive (cIOM); cIOMb, group b of cIOM; cIOMbe, group beta of cIOM; cIOMd,group d of cIOM; cIOMc, group c of cIOM; IODdf, dorsal fold of dorsal olive (IOD); IODvf, ventral fold of IOD; iIOM, intermediate part of medial olive (IOM); rIOM, rostral part of IOM; IODvf, ventral fold of IOD; IOPvf, ventral fold of principal olive (IOP); IOPdm, dorsomedial group of IOP; IOPdf, dorsal fold of IOP. Cbl-zone: sagittally oriented zones of Purkinje cells in the cerebellar cortex indicated by capital letters A to D followed by either a number or a lowercase ‘x’ and related to zebrin-positive or zebrin-negative stripes. CN: cerebellar nuclei from left to right: vMed, ventral part of medial cerebellar nucleus (Med); rMed, rostral part of Med; cMed, caudal part of Med; MedcDL, caudal part of dorsolateral protuberance of Med; MedrDL, rostral part of dorsolateral protuberance of Med; IntIC, interstitial cell groups; LV, lateral vestibular nucleus; IntA, anterior interposed nucleus; IntIC, interstitial cell groups; IntP, posterior interposed nucleus; IntA, anterior interposed nucleus; vLat, ventral part of lateral cerebellar nucleus (lat); IntDL, dorsolateral hump; dLat, dorsal part of the Lat. Based on data from Ruigrok [49], Fujita et al. [150] and Apps et al. [48]

As the layer V pyramidal cells constitute the main and excitatory output source of the cerebral cortex, cerebellar cortical output is governed by the monolayer of the GABAergic Purkinje cells. However, this cerebellar cortical output is mostly sluiced through the cerebellar nuclei and their output is directed to a multitude of targets which, at least to a considerable extent, can reach the cerebrum. Therefore, before dealing with the output of the cerebellum it is important to recognize the role of the organization of the corticonuclear connections. It is well known that relatively small groups of cerebellar nuclear cells receive input from Purkinje cells that are arranged in longitudinal strips that are aligned with the direction of their dendrites [26, 106]. The resulting anatomical corticonuclear organization relates to biochemical and physiological specificities observed within the Purkinje cells [48, 50]. As the corticonuclear organization matches the olivocortical and olivonuclear as well as nucleo-olivary organization [49, 107, 108], cerebellar functional operation has been suggested to be related to this modular organization [48]. In mice and rat at least 14 of these olivo-cortico-nuclear modules have been recognized [26], many of which may be further subdivided (see Fig. 5). In primates, the modular organization shows many similarities with the rodent system although some differences concerning the hemispheral parts of the crura have been noted [83, 109]. These differences to some extent also find their origin in the distinct relations between vermal and hemispheral lobules as observed between rodents and primates in the morphology and related nomenclature. An excellent review on this subject was provided by Sugihara [18].

The question now arises which of these cerebellar modules can influence the cerebrum, how they accomplish that, and what role they play in cerebral functioning and learning processes. A related question should deal with the functional potential of the recurrent cerebro-cerebellar circuitry [16].

### The cerebello-thalamo-cortical Connection

Cerebellar information mostly reaches the cerebral cortex by way of several thalamic intermediaries. Classically, the ventral lateral (VL) and ventral anterior (VA) thalamic nuclei, in rodents usually combined as the VA/VL complex, specifically connect to the primary and pre-motor cortices and are generally seen as the most significant targets for cerebellar output involved in motor control. However, more recently, other thalamic areas, such as the posterior and ventromedial nuclei, have also been implicated in motor control [13, 110, 111]. In addition, many regions of the cerebellar nuclei also supply many other thalamic areas, such as the mediodorsal and intralaminar nuclei [112–115]. Presently, it is not clear if these nuclei are also involved in motor control or in other cerebellar functions.

**Fig. 6:**
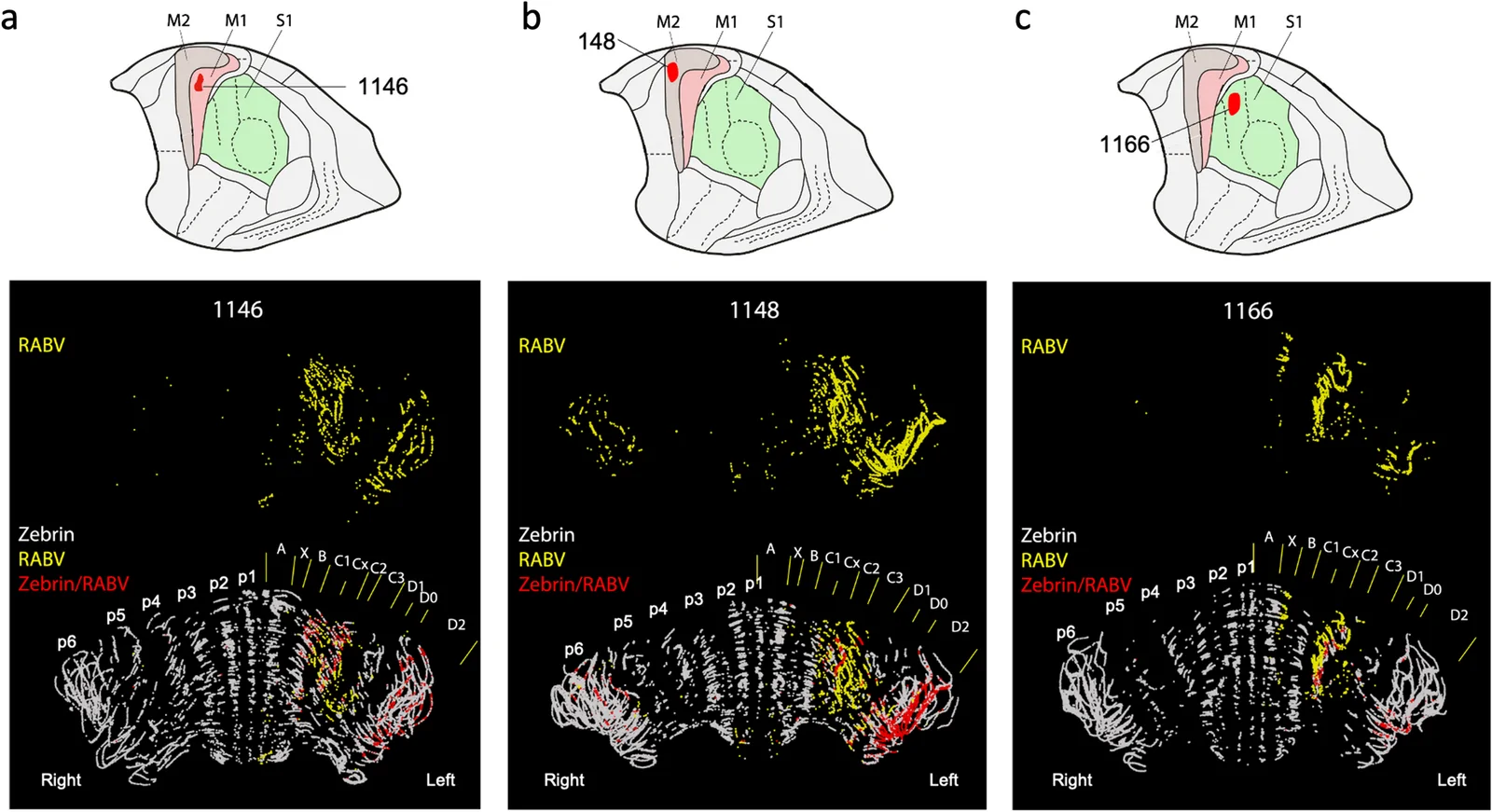
Trisynaptic cerebello-cerebral connections studied with the retrograde transneuronal infection with RabV in the rat. Top panels show the injection sites in a flattened representation of the right cerebral cortex. Bottom panels show rostral views of superimposed plots of RabV-infected Purkinje cells only (top diagram) or together with the zebrin-pattern of the Purkinje cells (bottom diagram) of coronal sections of the anterior part of the cerebellum. Zebrin-positive stripes are indicated p1-p6 and are used to delineate the Purkinje zones A to D2 as based on the diagram of Fig. 5. (**a**) Injection centered on the M1 region. (**b**) Injection centered on the M2 region. (**c**) Injection centered on the forelimb area of S1. Note the vermal, paravermal and hemispheral essentially longitudinal clusters of infected Purkinje cells in all three cases. Cerebello-cerebral connections are mostly contralateral (left side), but M2 also receives information from ipsilaterally located Purkinje cells. Both zebrin-positive (red dots) and zebrin-negative Purkinje cells (yellow dots in bottom diagram) supply information to the cerebral cortex

In macaque studies employing the retrograde transneuronal infection with unmodified rabies virus, Peter Strick and his group showed that the dorsal part of the dentate nucleus of the cerebellar nuclear complex are origin to nucleo-thalamo-cortical projections that reach the motor cortex [116]. More ventral dentate regions are directed to prefrontal and posterior parietal areas and are thought to subserve non-motor functions. This notion was confirmed in human in an fMRI study [117]. However, although in primates the dentate nucleus is clearly the largest, the other cerebellar nuclei also are supplying efferents to the thalamus and will evidently play their role in cerebellar involvement on aspects of motor control. In rodents, a retrograde transneuronal study with unmodified rabies virus injections in the M1, M2 and S1 area of the rat cortex invariably showed that infected Purkinje cells could be observed in vermal, paravermal and hemispheral parts of the cerebellar cortex (Fig. 6). This implies not only that a single cortical region as M1 or M2 receives input from several cerebellar modules, but also that the output of individual cerebellar modules would be directed to functionally different cortical areas as M1 and M2 and, indeed, although more sparsely, to S1 [118]. The notion of partially overlapping thalamic territories of different cerebellar nuclei has also been shown in primates [112] and suggests that multiple cerebellar modules may contribute to different aspects of the same movement [48, 49, 111]. It should be mentioned here, that within the modules, that stretch out over multiple cerebellar lobules, topographical differences can be observed related to the various somatotopic representations over the cerebellar cortex [15] and correlate with the somatotopy of the cerebral cortex [118].

### Cerebellar Connections with the Basal Ganglia

As the thalamic territories of basal ganglia output and cerebellar output not only are largely separated, but also make use of inhibitory and excitatory neurotransmitters, respectively [119–121] they have long been considered as separate entities in the control of movement. However, it has become quite clear that output of the cerebellar nuclei, by way of a thalamic link, can influence striatal processing [122, 123]. More recently, in conjunction with cerebellar output directed to the ventral tegmental area and substantia nigra [124, 125], these alternative cerebello-cerebral routes have been implicated in a cerebellar role in reward mechanisms [124, 125]). Furthermore, cerebellar subcortical interaction with the basal ganglia may affect the basal ganglia role in movement selection and initiation [126]. In addition, cerebellar dysfunction may result in typical basal ganglia disorders such as dystonia [122, 127].

A reverse disynaptic connection from basal ganglia, originating from the subthalamic nucleus to the cerebellar cortex has also been reported in primates [128] and rodents [129, 130]. The basal pontine nuclei are seen as the main intermediary in this connection [105, 129] and was confirmed with tractography in humans [131]. The connection may be specifically related to decision making processes as the cerebellar crus 1 and 2 regions are implicated as their main target. However, a cerebellar connection of subthalamic efferent fibers by way of the pedunculopontine region [132–134] cannot be ruled out [130] as a cholinergic projection to the cerebellar cortex partly finds its origin here [135]. Recently, a basal ganglia loop from the rodent entopeduncular nucleus (analogous with the primate internal globus pallidus) to the cerebellum has been suggested with the midbrain and inferior olive as intermediaries [136].

From this it is clear that the cerebellar and basal ganglia impact on motor behavior not only takes place in the motor and premotor cortices but that subcortical interactions between cerebellum and basal ganglia are implemented on their ultimate functional effect on motor control.

### Collateralization of Cerebellothalamic Projections to Brainstem and Spinal Cord

**Fig. 7:**
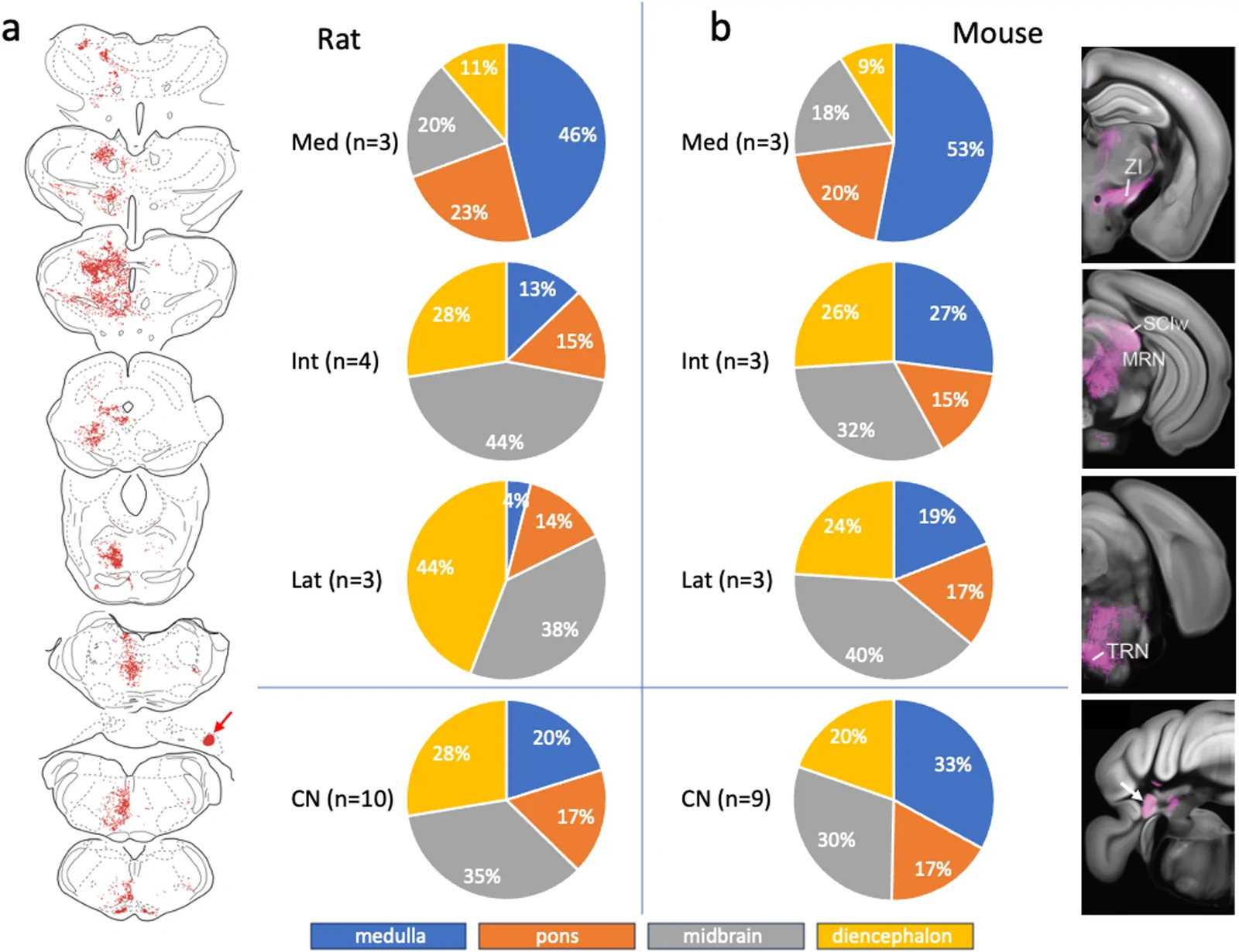
Comparison of the distribution of nucleo-bulbar projections in rat and mouse using anterograde tracing. (**a**) The number and location of terminal varicosities of labeled fibers after injections with either Phaseolus vulgaris leucoagglutinin (Pha-L) or biotinylated dextran amine (BDA) at various locations of the cerebellar nuclei was determined and averaged for the medial, interposed and lateral cerebellar nuclei (n numbers are indicated) as well as averaged over all nuclei (bottom). The result of a single injection (arrow) located in the dorsal part of the lateral nucleus is shown. (**b**) Similar for the mouse, but projections were now determined based on location and intensity of fluorescence after injection with an anterogradely transported adeno-associated virus (AAV9). A single example injection in the lateral nucleus (arrow) and resulting labeling is shown. Note that a single injection well located within part of the nuclei results in projections to large areas of the brainstem. In fact, in both rat and mouse most labeling is observed outside of the diencephalon. Abbreviations: CN, cerebellar nuclei; Int, interposed nuclei; Lat, lateral cerebellar nucleus; Med, medial cerebellar nucleus; MRN, midbrain reticular nucleus; SC, superior colliculus; TRN, tegmental reticular nucleus; ZI, zona incerta. Based on data from Teune et al. [115] and Hasanbegovic et al. [151]

**Fig. 8:**
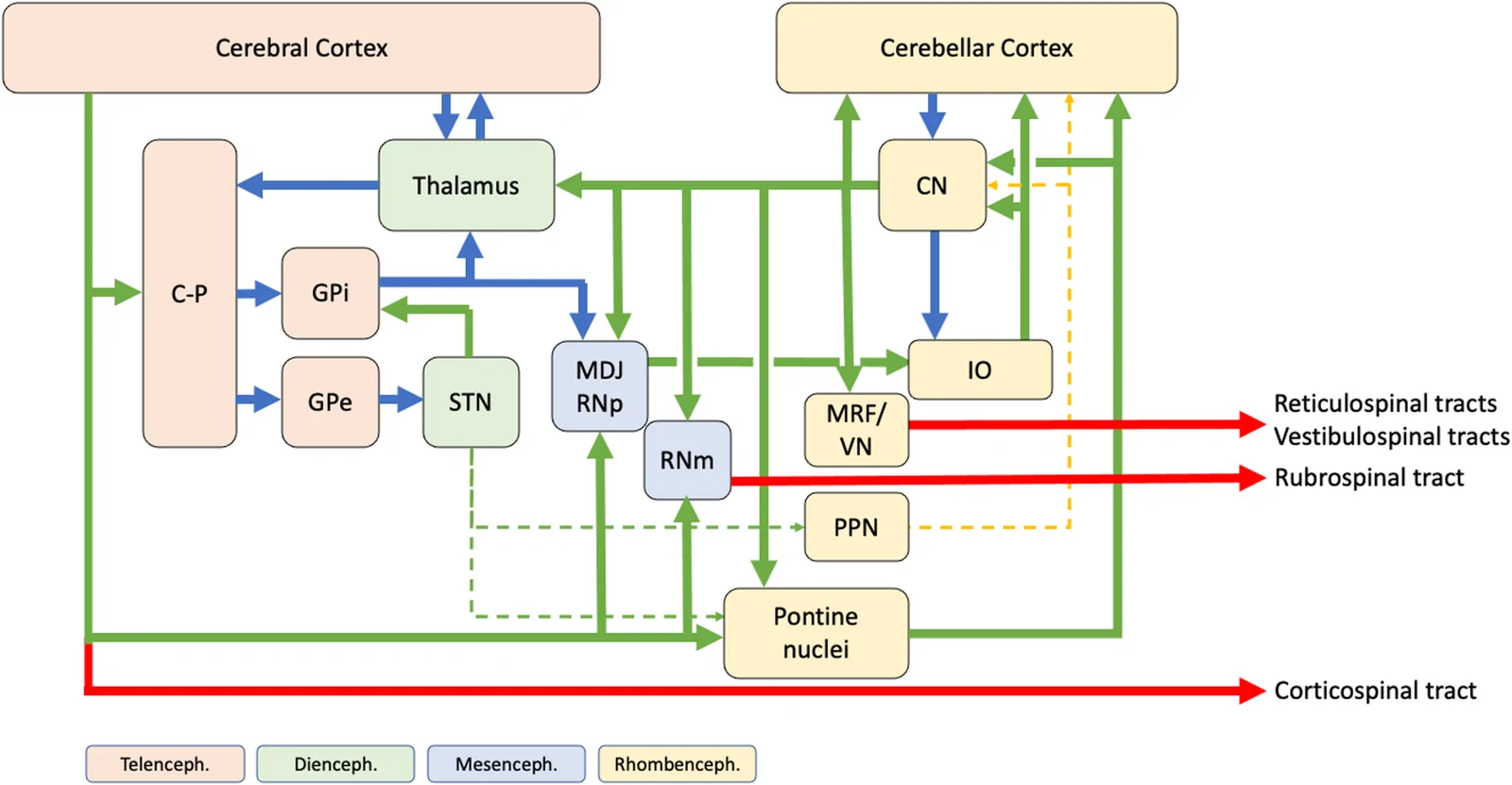
Simplified diagram of some cerebro-cerebellar connections and their intermediaries. Circuitries involving both the cerebral cortex and the basal ganglia are indicated. Note the multitude of potential loops involving both mossy fiber and climbing fiber circuits. Red arrows denote premotor connections, green arrows denote excitatory connections and blue arrows are inhibitory. Modulatory connections (cholinergic) are in orange Debated connections are indicated by hatching. Locations of the various brain regions are indicated by different shading colors. Abbreviations: CN, cerebellar nuclei; C-P, caudate-putamen; GPe, external part of globus pallidus; GPi, internal part of globus pallidus; IO, inferior olive; MDJ, mesodiencephalic junction; MRF, medial reticular formation; PPN, pedunculopontine nucleus; RNm, magnocellular part of the red nucleus; RNp, parvicellular part of the red nucleus; STN, subthalamic nucleus; VN, vestibular nuclei

Apart from the cerebellar impact on the (pre-)motor cortices and basal ganglia, another aspect should be taken into account when studying cerebellar control of movement. It is well-known that many cerebellar nuclear fibers that reach the thalamus also provide collaterals to many other areas in the brainstem and spinal cord [113, 115, 137], many of which, such as the magnocellular red nucleus and the medullary reticular formation, are thought to be involved in motor functions [138–141]. Furthermore, thalamus-directed cerebellar nuclear efferents also collateralize to precerebellar nuclei, such as the pontine nuclei or even directly to the cerebellar cortex [137, 142–144]. Indeed, when examining the quantitative distribution of terminals, data from rodents suggest that more terminals from the cerebellar nuclei terminate in the brainstem than in the thalamus (Fig. 7). These circuits are further supplemented by the general cerebro-cerebellar connections outlined above and direct connections between the cerebellar nuclei and the inferior olive [145], which are mostly, but not exclusively GABAergic [146]. Hence, it will be important to carefully dissect and evaluate the roles of cerebellar nuclear output in order to get a handle on the integrated function of the various recurrent pathways by way of direct nucleo-cortical, nucleo-precerebellar mossy fiber, nucleo-midbrain-olivary pathways, in conjunction with their impact on premotor systems such as the medial reticulospinal, rubrospinal and pyramidal tracts (Fig. 8).

## Conclusion

This review shows that available anatomical information on the connectivity between cerebral cortex and cerebellum may help to describe and understand general network functionality of the joint cerebro-cerebellar system in aspects of motor control. However, in order to fully grasp the intricacies of the cerebral cortico-cerebellar circuit, the impact of convergence and divergence of information streams, together with the functional importance of the various recurrent loops, as well as of the subcortical interactions between basal ganglia and cerebellum, more detailed information seems to be required. Simultaneously however, it should be recognized that evolutionary as well as developmental restraints may have resulted in several levels of redundancy in this system, the role of which is far from clear. Finally, as the cerebellum is also involved in cognitive, affective and autonomic aspects, both the structure and nature of these intertwined circuits may vary dependent on the specific ultimate function required by the nervous system.

## Data Availability

All data supporting earlier unpublished findings that are presented in this review are available upon request .
